# Breast Squamous Cell Carcinoma Following Breast Augmentation

**DOI:** 10.7759/cureus.3405

**Published:** 2018-10-03

**Authors:** Yu M Zhou, Huma E Chaudhry, Amar Shah, Janna Andrews

**Affiliations:** 1 Radiation Oncology, University of Cincinnati College of Medicine, Cincinnati, USA; 2 Radiation Oncology, Hofstra Northwell School of Medicine, Hempstead, USA; 3 Radiology, Hofstra Northwell School of Medicine, Hempstead, USA; 4 Radiation Oncology, Northwell Health, Sleepy Hollow, USA

**Keywords:** squamous cell carcinoma, breast implant related problems/revisions

## Abstract

Breast augmentation is the most common cosmetic surgery in the United States. Squamous cell carcinoma (SCC) of the breast raises suspicion of possibly metastatic origin. Here, we report an unusual case of implant-associated SCC of the breast post silicone gel breast implant. The patient is a 46-year-old female with SCC of the breast. She initially had silicone gel breast implantation for breast augmentation in 1995. She had multiple revisions due to swelling and hardening. In 2016, she underwent bilateral prosthesis explantation and bilateral capsulectomy. The pathology demonstrated a 4-cm tumor that was moderately differentiated invasive SCC. On slide review, it was noted that there was squamous epithelization of the implant capsule with benign squamous epithelium on both sides. She received external beam radiation to the right breast; no adjuvant chemotherapy was offered due to the rare histology and paucity of data. Follow-up within a year showed metastasis to the liver, lungs and retroperitoneum. She was admitted and ultimately transferred from the medical intensive care unit to the palliative care unit for comfort care. She expired of her disease in July 2017.

## Introduction

Breast augmentation is the most common cosmetic surgery in the United States. While local complications and reoperations are common, it is rare for silicone implants to be associated with conventional carcinoma [1]. Primary squamous cell carcinoma (SCC) of the breast is a rare and generally aggressive disease, representing less than 0.1% of invasive breast carcinomas [2]. SCC of the breast raises suspicion of possibly metastatic origin. Here, we report an unusual case of implant-associated SCC of the breast post silicone gel breast implant capsule.

## Case presentation

**Figure 1:**
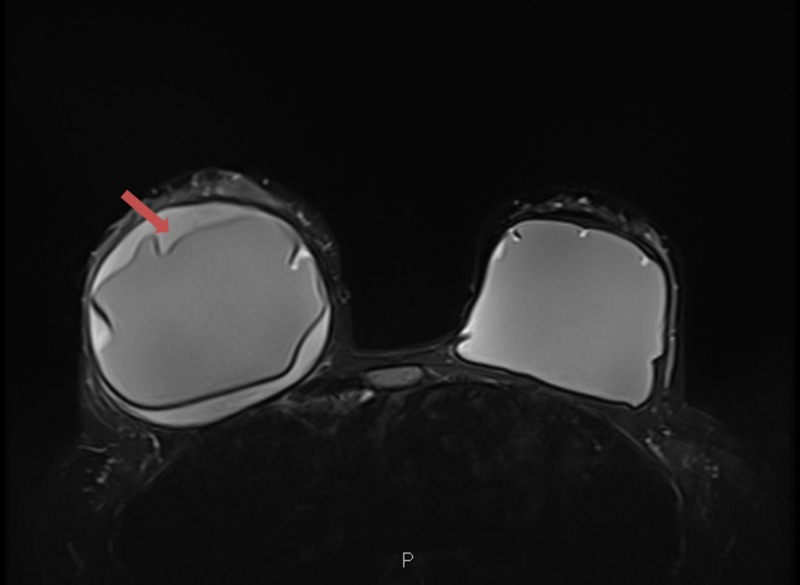
Bilateral breast magnetic resonance imaging (MRI). There is a large fluid collection, indicated by the red arrow, surrounding the intact right silicone implant. The fluid is interposed between the fibrous capsule and the implant.

The patient is a 46-year-old female with SCC of the breast. She initially had silicone gel breast implantation for breast augmentation in 1995. The implantation was surgically revised in 2002 and 2006. In 2014 she noticed hardening and swelling of her right breast. Because of the death of her husband, she did not seek immediate medical attention. She continued to have swelling and increased pain in the right breast. Magnetic resonance imaging (MRI) performed in January 2016 showed a large fluid collection surrounding the intact right silicone implant as shown in Figure 1. The case was managed by surgical drainage of fluid collection and capsulectomy. In February 2016, she underwent bilateral prosthesis explantation and bilateral capsulectomy. The pathology demonstrated a 4-cm moderately differentiated invasive SCC. It extended into the muscle, and in situ tumor was noted to extend to the peripheral margin. There was no perineural or lymphovascular invasion. Pathology of the left breast capsule showed chronic inflammation. Computed tomography (CT) of chest, abdomen and pelvis on February 2016 revealed absence of metastatic disease. The patient underwent re-excision of the remaining chest wall mass in March 2016. Pathology showed well differentiated SCC with negative margins. Chest wall fluid was negative for malignant cells. On slide review, it was noted that there was squamous epithelialization of the implant capsule with benign squamous epithelium on both sides. This indicated that the tumor is likely SCC of the implant capsule rather than primary SCC of the breast. Estrogen and progesterone receptor markers were negative as well. Afterwards, she received external beam radiation. She was treated with radiation while supine with free breathing. Four tangent beams were used to target the right breast with 50 Gray in 25 fractions, followed by a 10 Gray boost to the tumor bed delivered in five fractions. Radiation was delivered using opposed tangents completed in May 2016. No adjuvant chemotherapy was offered due to the rare histology and paucity of data. She followed up in clinic in June 2016 without complications or clinical recurrence.

**Figure 2:**
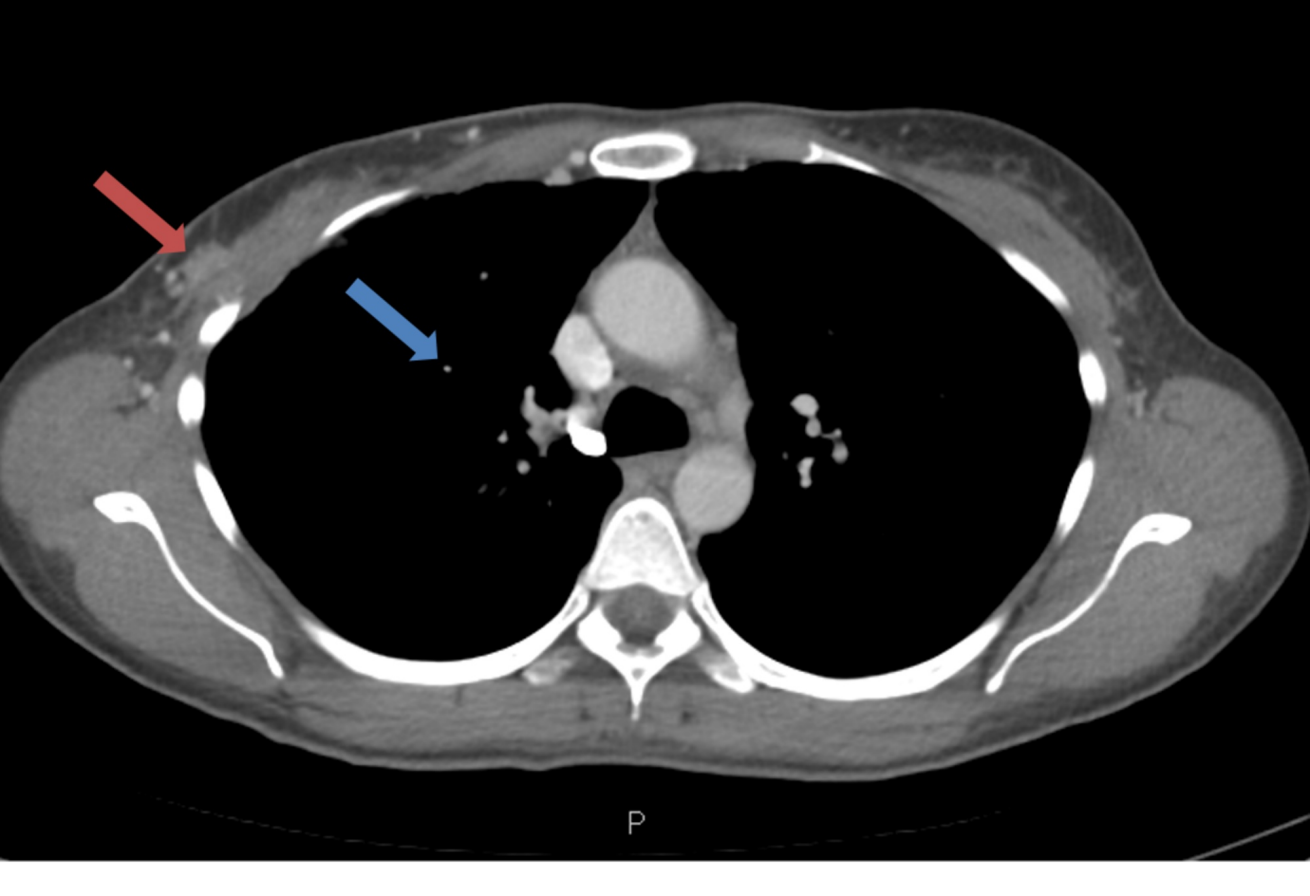
Computed tomography (CT) of the chest with intravenous and oral contrast. CT of the chest showing 1) 8 mm right upper lobe nodule suspicious for metastatic disease and 2) 6 x 12 mm enhancing area in lateral aspect of right breast which may be residual or recurrent disease.

**Figure 3:**
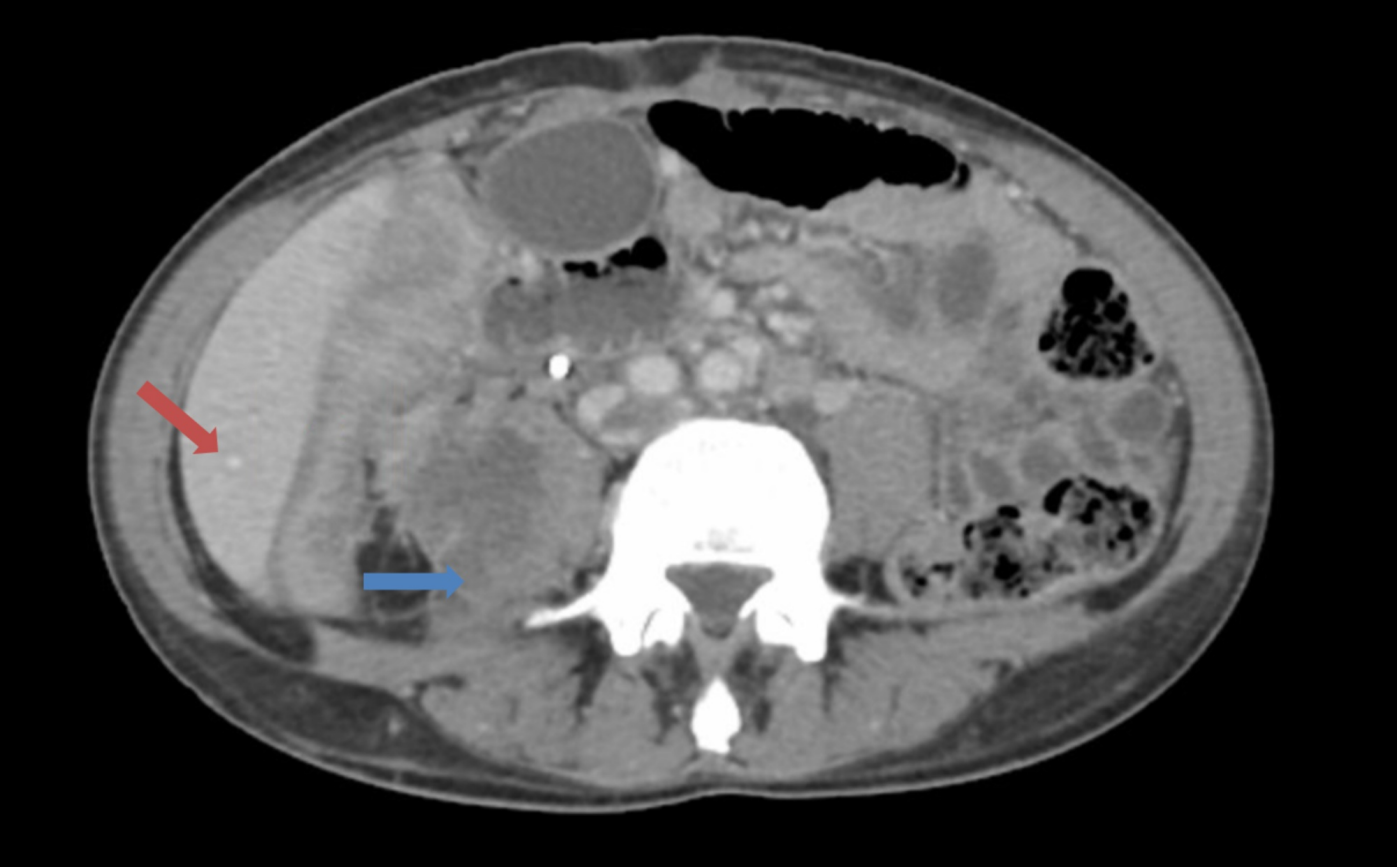
Computed tomography (CT) of abdomen showing multiple metastasis. CT image showing 1) multiple new hepatic lesions, likely metastases (red arrow) and 2) heterogeneous lesion involving the lower pole of the right kidney with extension into the right psoas muscle (blue arrow).

Follow-up CT scan performed in August 2016 displayed a right upper lobe lung nodule and findings were suspicious for local recurrence (Figure 2). She underwent right video thoracoscopy and right upper lobe wedge resection. The pathology was consistent with metastatic moderately differentiated SCC. The patient declined chemotherapy at this time. CT chest and abdomen at another hospital showed new cavitary lung nodules and right renal and psoas abscess. In February 2017, retroperitoneal fine needle aspiration of the right renal collection was positive for SCC. In June 2017, she was admitted to the hospital for abdominal pain and was found to have progressive disease. CT abdomen and pelvis with intravenous and oral contrast on 6/16/17 demonstrated a 6.1 cm x 5.7 cm heterogeneous lesion in the right kidney lower pole with invasion into the adjacent right psoas muscle (Figure 3). Progressive metastases to the liver, lungs and retroperitoneum were noted as well. Ultrasound-guided fine-needle aspiration and core biopsy of the liver was positive for metastatic SCC with keratinization and necrosis.

Her hospital course was complicated by non-ST elevation myocardial infarction, recurrent anemia requiring transfusions, atrial fibrillation with rapid ventricular rate and hypotension. She was noted to have leptomeningeal spread. She was ultimately transferred from the medical intensive care unit to the palliative care unit for comfort care. She expired of her disease in July 2017, one year after her initial diagnosis of cancer.

## Discussion

Squamous cell carcinoma of the breast is a rare diagnosis. Data from the National Cancer Institute’s Surveillance, Epidemiology and End Results Registry showed a total of 445 cases between the year 1998 and 2013, with age adjusted incidence of 0.62 per 1,000,000 per year [3]. From the registry, majority of the patients were triple negative for breast markers. Over half of the patients underwent mastectomy and one-third of the cases received radiation. The five-year cause-specific survival was 63.5%.

The available literature shows only five cases of SCC arising from breast implant surrounding capsular tissue [4-7]. In the reported cases as well as in the current patient, there is a remote history of breast augmentation (>15 years) with silicone breast implants as shown in Table 1. All patients had an insidious onset of breast pain and many initially were misdiagnosed. Since three out of the four patients in the literature with recorded follow-up had multiple metastatic disease within two years, this may suggest the aggressive course of the tumor and an inadequacy of local control. The presence of widespread multiple metastases post-surgery and radiation highlights the importance of incorporating systemic therapy. Early diagnosis may be the key to improving disease-specific survival. In previously reported cases, most patients underwent negative sentinel lymph node biopsies. The observation that some patients deteriorated rapidly may suggest a more aggressive hematogenous metastasis mechanism which is more consistent with SCCs.

**Table 1:** Comparison of case studies. Comparison of presentation, pathology and outcomes of breast implant associated SCC [4-7]. CRT: Chemoradiation; *: Pathology associated with implant capsule; NR: Not reported; SCC: Squamous cell carcinoma.

	Paletta et al. (1992) [4]	Satgunaseelan et al. (2014) [5]	Zomerlei et al. (2015) [6]	Olsen et al. (2017) [7]	Olsen et al. (2017) [7]	Zhou et al. (2017)
Age at diagnosis (years)	52	58	58	56	81	46
Time of initial implant	1970s	1985	1980s	1984	1970s	1995
Initial onset of symptoms	1992: pain and induration	2014: pain and induration	2015: pain and induration	2012: pain and induration	2012: pain and induration	2014: pain and induration
Pathology	Moderately differentiated SCC	High grade SCC	Well differentiated SCC*	Well-moderately differentiated SCC*	Well-moderately differentiated SCC*	Well-moderately differentiated SCC*
Hormone status: ER, PR, Her2/neu	NR	NR	Triple negative	Triple negative	Triple negative	Triple negative
Sentinel lymph nodal status	NR	NR	Negative	Negative	Negative	Negative
Management	Mastectomy	Mastectomy	Bilateral mastectomies	Mastectomy then CRT	Mastectomy then CRT	Excision and radiation
Follow up	No evidence of disease	NR	NR	Multiple metastasis	Multiple metastasis	Multiple metastasis
Follow up duration	One year	NR	NR	Two years	One year	One year

According to the World Health Organization (WHO) classifications of breast tumors, diagnosis of primary squamous cell carcinoma requires: (1) more than 90% of the malignant cells must have squamous differentiation, (2) there are no other primary sites of SCC, and (3) the lesion must be separate from the skin and nipple [8]. In all cases, the patient had no clinical or radiologic evidence of primary SCC of the breast or cutaneous or distant invasive SCC, as such the reported cases have met these criteria. As with most SCC, the tumors were negative for breast markers, suggesting that these tumors are resistant to hormone treatments.

In this paper, we present a rare case of SCC of the breast with a rare mechanism of onset. Our patient had no evidence of primary mammary carcinoma or SCC from other sites. Pathology report on initial excision of the tumor supports the origin of the tumor as squamous epithelialized implant capsule. However, the source of the epithelial cancer is unclear. Multiple case studies, in addition to the previous discussed case reports, have suggested silicone placement can lead to metaplastic squamous epithelium which may be a precursor to SCC [9]. Metaplasia can occur in general post-surgically with presence of chronic inflammation such as Marjolin’s ulcers [10]. Although the patient’s left breast did not reveal any dysplasia, pathologic analysis did show signs of chronic inflammation. The findings in the non-cancerous left breast suggest a chronic inflammatory process which may take 10 to 20 years for epithelial to turn dysplastic. However, the source of the epithelial cancer remains unknown.

Our patient’s course of rapid hematological spread highlights both the aggressive nature of disease and its poor prognosis. Given the aggressiveness of the disease, there may be a need for a different screening protocol for patients with breast implants. There is a difference in outcomes in our comparison of the case studies. Olsen et al. were the first to report a rapid progression of the disease with poor prognosis. In other reported cases, follow-ups were either not reported or showed no disease progression. The dichotomy of outcomes shows the need for close follow-up to better understand the disease.

## Conclusions

There should be a high index of suspicion for SCC of the breast in patients with a history of breast implants, multiple post-implant complications and insidious onset of breast pain and induration. If the diagnosis of SCC is confirmed, aggressive systemic therapy may be needed to improve future outcomes.
